# Effect of Transcatheter Aortic Valve Implantation on Non-Invasive Myocardial Work Parameters: A Systematic Review and Meta-Analysis

**DOI:** 10.3390/jcm14196997

**Published:** 2025-10-02

**Authors:** Isabella Leo, Federico Sicilia, Jolanda Sabatino, Angelica Cersosimo, Nicole Carabetta, Antonio Strangio, Giuseppe Panuccio, Giovanni Canino, Jessica Ielapi, Nadia Salerno, Sabato Sorrentino, Daniele Torella, Salvatore De Rosa

**Affiliations:** 1 Department of Experimental and Clinical Medicine, Magna Graecia University, 88100 Catanzaro, Italy; isabella.leo@unicz.it (I.L.); federico.sicilia@studenti.unicz.it (F.S.); angelica.cersosimo@unicz.it (A.C.);; 2 Research Center for Cardiovascular Science, Magna Graecia University, 88100 Catanzaro, Italy; 3 Pediatric Research Institute (IRP) ‘Città della Speranza’, 35127 Padua, Italy; 4 Department of Women’s and Children’s Health, University Hospital Padua, 35128 Padua, Italy; 5 Department of Medical and Surgical Sciences, Magna Graecia University, 88100 Catanzaro, Italy

**Keywords:** aortic stenosis, transcatheter aortic valve implantation, non-invasive myocardial work evaluation, global longitudinal strain, speckle tracking echocardiography

## Abstract

**Background/Objectives**: Aortic stenosis (AS) leads to progressive left ventricular (LV) pressure overload, adverse myocardial remodeling, and eventual functional decline. While traditional parameters such as left ventricular ejection fraction (LVEF) may remain preserved until advanced stages, they are insufficiently sensitive to early dysfunction. Global longitudinal strain (GLS) offers improved detection but remains load-dependent. In contrast, non-invasive myocardial work (MW)—derived from pressure-strain loops—offers a more load-independent assessment of myocardial function. This systematic review and meta-analysis aimed to evaluate the effects of transcatheter aortic valve implantation (TAVI) on MW indices in patients with severe AS. **Methods**: We performed a systematic review and meta-analysis of studies reporting non-invasive myocardial work parameters before and after TAVI (PROSPERO ID: CRD420250517138). Databases were searched through 31 March 2025. Pooled mean differences in global work index (GWI), global constructive work (GCW), global wasted work (GWW), and global work efficiency (GWE) were calculated using random-effects models. Sensitivity analyses and meta-regression were conducted to explore heterogeneity and the influence of baseline characteristics. **Results**: Eleven studies encompassing 1493 patients were included. TAVI was associated with a significant reduction in GWI (−236.67 mmHg% [95% CI: −373.82 to −99.52]; I^2^ = 97.0%; *p* = 0.002) and GCW (−243.71 mmHg% [95% CI: −407.38 to −80.03]; I^2^ = 97.4%; *p* = 0.006). No significant changes were observed in GWW or GWE. Meta-regression showed age and baseline LVEF significantly influenced GWE changes, but not other parameters. **Conclusions**: TAVI leads to a significant reduction in GWI and GCW, reflecting decreased myocardial workload and afterload relief. These findings support the utility of MW indices as valuable tools for assessing myocardial adaptation post-TAVI and potentially guiding clinical decision-making.

## 1. Introduction

Aortic stenosis (AS) represents one of the most prevalent valvular heart diseases in the elderly population, with a prevalence of severe AS up to 3–5% in individuals over 75 years [1]. The presence of AS results in chronic left ventricular (LV) pressure overload that, after an initial compensatory LV hypertrophy, eventually leads to adverse remodeling, impaired systolic and diastolic function [2]. Transcatheter aortic valve implantation (TAVI) has transformed the therapeutic landscape for patients with severe AS, offering significant survival benefits and improved clinical outcomes even among high-risk populations unsuitable for surgical approaches [3]. Despite these advances, residual myocardial damage following valve replacement remains a clinical concern, as well as the optimal timing for intervention. Traditional echocardiographic indices such as LV ejection fraction (LVEF) have limitations in accurately depicting myocardial performance in AS due to their load dependence. Speckle tracking-based indices have proved to be able to identify early myocardial changes even in this subset of patients, although they are load-dependent parameters with possible limited accuracy in this setting. To overcome these limitations and provide a more load-independent assessment of myocardial function, a non-invasive estimation of myocardial work (MW) has been developed and recently validated [4], using an integration of LV strain analysis and a non-invasive estimation of LV pressure [5]. Recent studies have suggested that MW indices, including global work index (GWI), global constructive work (GCW), global wasted work (GWW), and global work efficiency (GWE), offer additional prognostic value beyond conventional measures by accurately reflecting myocardial deformation and function before and after TAVI. However, there is still variability and limited consensus regarding how these indices change post-TAVI.

Aim of this paper was to conduct a systematic review and meta-analysis to evaluate the effects of TAVI on non-invasive MW parameters in patients with severe aortic stenosis.

## 2. Methods

This meta-analysis was performed according to the Preferred Reporting Items for Systematic Reviews and Meta-Analyses (PRISMA) guidelines [6] (Table S1). The protocol has been published in the PROSPERO International prospective register of systematic reviews (CRD420250517138).

### 2.1. Search Strategy

Two independent investigators (A.C. and F.S.) performed a comprehensive literature search in PubMed, ClinicalTrials.gov, Embase, and the Cochrane Library using the following search terms: (“myocardial work “OR” pressure-strain loop” AND (“aortic stenosis” OR “aortic valve stenosis”) AND (“TAVI” OR “TAVR” OR “transcatheter aortic valve implantation” OR “transcatheter aortic valve replacement”) in various combinations. Full-text manuscripts published between 1 January 2010 and 31 March 2025 were screened for eligibility.

### 2.2. Study Eligibility

Full-text manuscripts published in peer-reviewed journals reporting baseline and post-TAVI non-invasive MW indices were included. Non–English-language studies, editorials, letters, expert opinions, case reports or series, duplicated data were excluded. No sample size restrictions were applied. Two authors (A.C. and F.S.) independently evaluated studies for eligibility, and discrepancies were resolved by a third reviewer (I.L.). Only studies that met all inclusion criteria were included in the final analysis.

### 2.3. Data Extraction

The following variables were collected: (i) first author, (ii) year of publication, (iii) study design, (iv) sample size, (v) main demographic, clinical baseline patient characteristics. Main echocardiographic parameters were also collected and included left ventricular ejection fraction (LVEF), global longitudinal strain (GLS), aortic valve (AV) mean gradient, aortic valve area (AVA), left atrium volume index (LAVi), global work index (GWI), global constructive work (GCW), global wasted work (GWW) and global work efficiency (GWE). Non-invasive MW was calculated using the dedicated GE (Healthcare, Chicago, IL, USA) software. A minimum of three studies reporting non-invasive MW outcome variables before and after TAVI was required for inclusion in the analysis. The individual quality of each study was assessed using the Newcastle–Ottawa Scale (NOS), with studies categorized as poor, fair, or good quality based on criteria related to selection, comparability, and outcome [7] (Table S2). Risk of bias for the included studies was assessed using the ROBINS-I tool, and the results are illustrated in the traffic-light plots provided in Table S3 [8].

### 2.4. Statistical Analysis

The primary endpoints of this analysis were the differences in the mean changes in key MW parameters between baseline and follow-up evaluations after TAVI. A random-effects model (DerSimonian and Laird method [9]) was applied to calculate pooled mean differences and corresponding 95% confidence intervals (CIs) for non-invasive MW parameters before and after TAVI. The effect size was defined as the mean change in the outcome of interest for each study, while the standard error (SE) of the mean difference was calculated from the reported change in standard deviation (SD) and sample size. When SDs were not directly reported, they were imputed based on available information according to Cochrane Handbook recommendations [10], using available confidence intervals, *p*-values from parametric tests of change, or from correlation coefficients. If not provided in the study, correlation coefficients were either extracted or imputed based on data from similar studies.

Heterogeneity was assessed using Cochran’s Q test and quantified using the I^2^ statistic, with values exceeding 50% interpreted as indicative of substantial heterogeneity [11]. Publication bias was evaluated through visual inspection of funnel plot asymmetry and formally tested using Egger’s regression intercept, with a *p*-value < 0.10 considered suggestive of significant asymmetry.

A leave-one-out analysis was performed by sequentially excluding one study at a time to identify potential sources of heterogeneity. Meta-regression analyses were also conducted to explore potential sources of heterogeneity and assess the influence of covariates on the observed effect sizes. All statistical analyses were performed using JASP (University of Amsterdam, v. 0.19.3), and a two-tailed *p*-value < 0.05 was considered statistically significant.

## 3. Results

### 3.1. Literature Search

**Figure 1:**
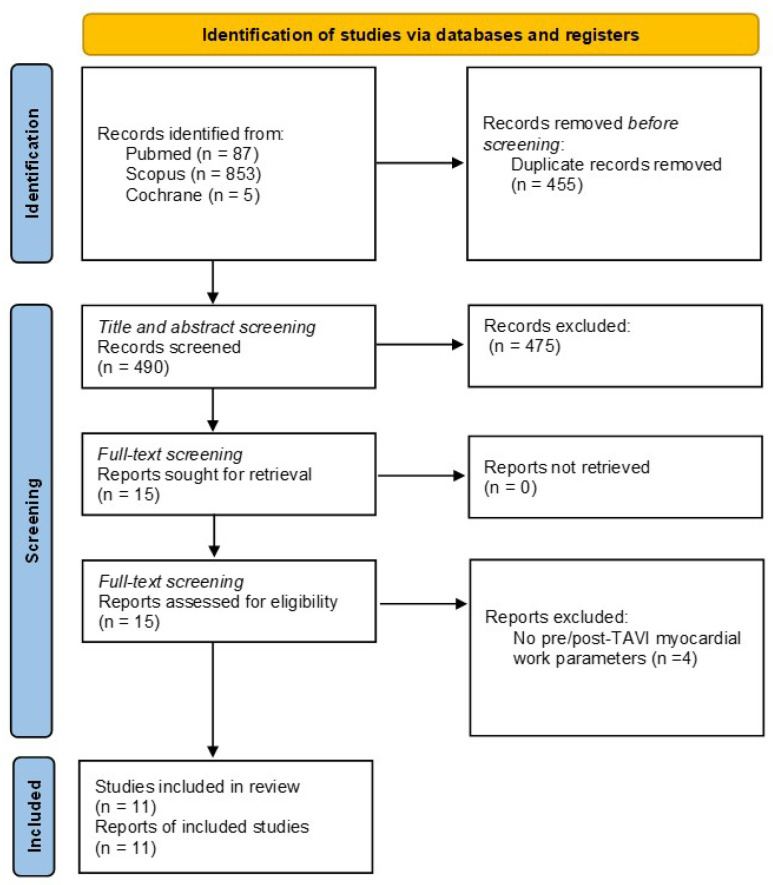
Study screening flow diagram.

The study flow-chart is reported in Figure 1. Initially, 945 articles were identified, with 455 duplicates removed. After screening the titles and abstracts of 407 articles, 15 were selected for full-text evaluation. Ultimately, 11 articles were judicated eligible for quantitative analysis of TAVI effects on MW [12,13,14,15,16,17,18,19,20,21,22] (Table 1).

### 3.2. Study Characteristics

A total of 11 studies including 1493 patients undergoing TAVI undergoing baseline and follow-up echocardiography were selected for quantitative analysis. The mean age ± SD of the included patients was 80 years ± 7. The median echocardiographic follow-up time was 52 days. Main baseline and echocardiographic characteristics are summarized in Table 1 and Table 2.

The smallest study had a population of 35 patients [15] and the largest 473 [19]. Overall, 7 studies [12,14,16,17,18,19,20] included analysis for low-flow low-gradient severe AS. Pedersen et al. [19] reported data on 6 subgroups based on the subtype of AS (high-gradient, low-flow low-gradient, normal-flow low-gradient) and according to baseline LVEF (>50%, <50%;); this has been included in the analysis per subgroups, according to how data were reported in the main manuscript.

### 3.3. Meta-Analyses

**Figure 2:**
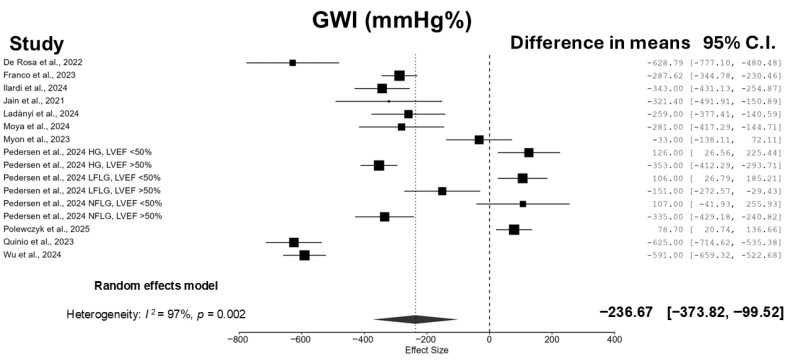
Forest plot summarizing the results of the meta-analysis evaluating the effect of TAVI on GWI. Data from 11 studies are included in the pooled estimate [12,13,14,15,16,17,18,19,20,21,22].

**Figure 3:**
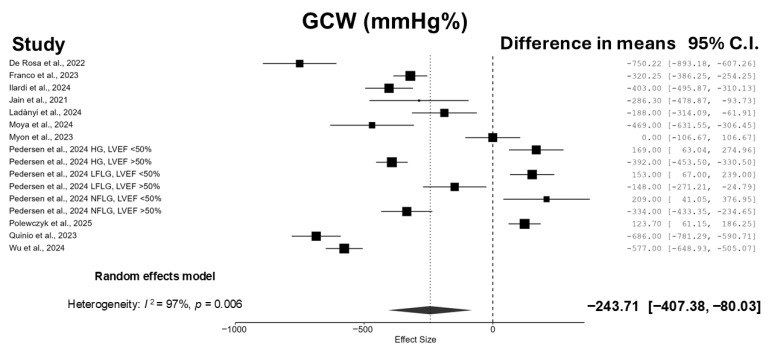
Forest plot summarizing the results of the meta-analysis evaluating the effect of TAVI on GCW. Data from 11 studies are included in the pooled estimate [12,13,14,15,16,17,18,19,20,21,22].

**Figure 4:**
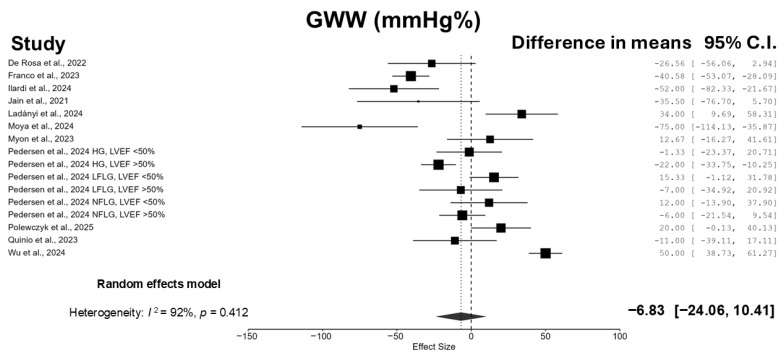
Forest plot summarizing the results of the meta-analysis evaluating the effect of TAVI on GWW. Data from 11 studies are included in the pooled estimate [12,13,14,15,16,17,18,19,20,21,22].

**Figure 5:**
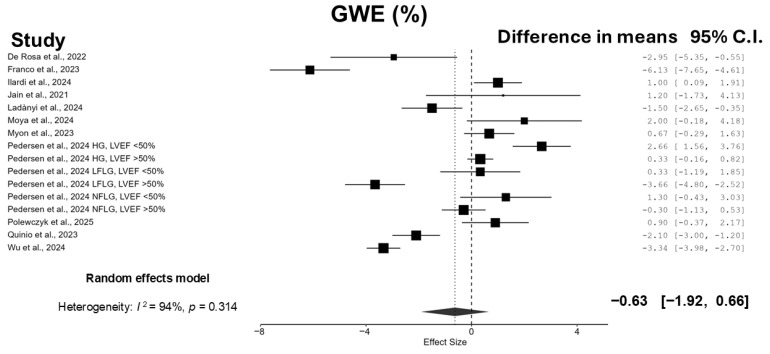
Forest plot summarizing the results of the meta-analysis evaluating the effect of TAVI on GWE. Data from 11 studies are included in the pooled estimate [12,13,14,15,16,17,18,19,20,21,22].

Patients who underwent TAVI experienced a significant reduction in GWI (−236.67 mmHg% [95% CI: −373.82, −99.52]; I^2^ = 97.0%; *p* = 0.002) (Figure 2) and GCW (−243.71 mmHg% [95% CI: −407.38, −80.03]; I^2^ = 97.4%; *p* = 0.006) (Figure 3), whereas no significant changes were observed in GWW (−6.83 mmHg% [95% CI: −24.06, 10.41]; I^2^ = 91.6%; *p* = 0.412) (Figure 4) and GWE (−0.63% [95% CI: −1.92, 0.66]; I^2^ = 94.0%; *p* = 0.314) (Figure 5).

### 3.4. Sensitivity Analysis

The robustness of the meta-analytic estimates across all non-invasive MW parameters was assessed by leave one-out analyses (Table S4). For GWI, pooled effect estimates ranged from −327.93 mmHg% to −211.62 mmHg%, always maintaining statistical significance (*p*-values ranging from 0.001 to 0.005), while heterogeneity remained high across all analyses, ranging from 96.3% to 97.3%. Similarly, analyses conducted on GCW did not reveal any significant effects of individual studies on the pooled effect estimates (ranging from −354.46 to −210.69 mmHg%), statistical significance (all *p* values < 0.05) or heterogeneity (96.8–97.6%). We did not identify any study that significantly impacted meta-analytic results for both GWE and GWW.

### 3.5. Meta-Regression Analyses

Meta-regression analyses showed that none of the covariates (age, LVEF, percentage of women) significantly moderated the effect size for GWI, GCW, or GWW. In contrast, both mean age (*p* = 0.041) and mean LVEF (*p* = 0.020) were significant moderators of GWE changes.

### 3.6. Publication Bias and Grading of Evidence

**Figure 6:**
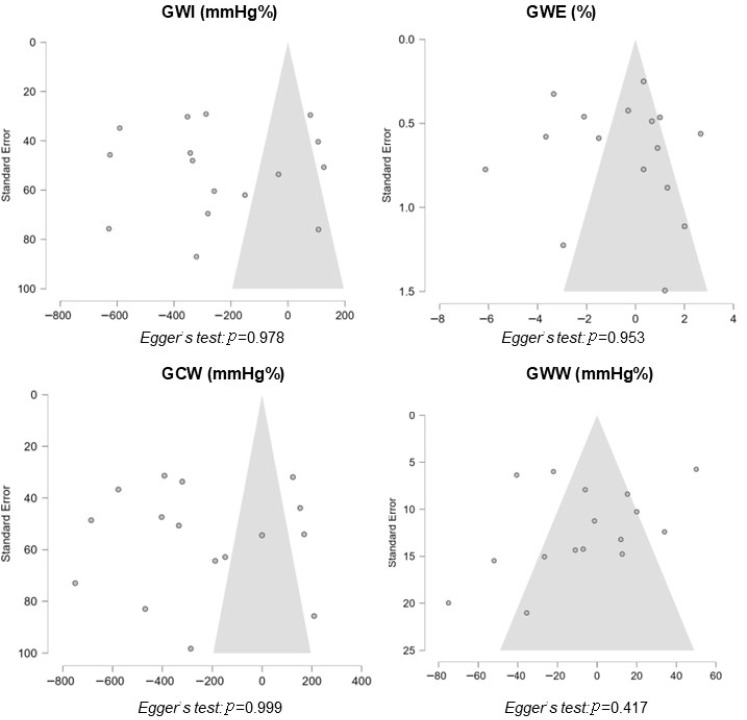
Evaluation for publication bias. Funnel plots with 95% confidence intervals for GWI, GWE, GCW and GWW.

Potential publication bias was assessed through visual inspection of funnel plots and Egger’s regression test for all MW parameters. The Egger’s test did not detect any significant funnel plot asymmetry for any of the outcomes, suggesting no evidence of publication bias (Figure 6). According to the GRADE Working Group system [23], the level of certainty for the association between TAVI treatment and MW outcomes was adjudicated very low for all outcomes (Table 3).

## 4. Discussion

The present systematic review and meta-analysis demonstrated that TAVI is associated with significant reductions GWI and GCW, while no significant change was noted for GWW and GWE. These findings reinforce the data already present in the literature about the role of non-invasive MW indices in this clinical setting. The prognostic value of echocardiographic markers in patients undergoing interventional procedures is well established [24], while advanced echocardiography has already demonstrated to overcome some of the limitations of traditional echocardiographic parameters [25]; in patients with severe AS, this is of utmost importance given the limitations of LVEF as accurate marker of systolic function. The chronic LV overload leads to LV hypertrophy and reduced LV volumes which in turns are responsible for an initial increase in LVEF to preserve cardiac output [26]. The decrease in LVEF is a late marker of disease, associated with the presence of irreversible myocardial damage. The recently published 2025 ESC guidelines highlighted the importance of advanced imaging modalities and a multiparametric approach to optimize timing of intervention in AS [27]. In particular, GLS is now recognized as a sensitive marker of early LV systolic dysfunction, with a cutoff of −15% that may help in detecting severe asymptomatic AS patients at elevated risk of subsequent clinical deterioration or early death [28]. Myocardial work indices by integrating strain with afterload may offer incremental value beyond GLS, providing a more comprehensive and afterload-independent measure of myocardial performance. In future, these indices may be used to guide timing of intervention in asymptomatic patients with important repercussion on prognosis and long-term outcomes. TAVI has been widely demonstrated to improve symptoms, functional status, and survival in patients with severe AS [29,30], but despite successful valve replacement and hemodynamic correction, residual myocardial damage may persist and negatively impact long-term outcomes [31]. Global work index and GCW are different markers of systolic function, reflecting the cumulative work during systole (GWI) and the positive work performed by the myocardium during systolic shortening and isovolumic relaxation lengthening (GCW) [32]. Both these parameters are typically increased in the early phase of the disease because of elevated afterload, but gradually decrease as fibrosis progresses and myocardial functional reserve diminishes [33,34,35]. The observed post-TAVI reduction in GWI and GCW can be interpreted as a physiological response to the relief of increased afterload and myocardial oxygen demand after resolution of the mechanical obstruction [12]. This may also reflect an incomplete functional recovery after TAVI, that while offering immediate hemodynamic relief and symptomatic benefit does not completely restore the subclinical myocardial damage and the reverse remodeling [29,34]. Although more studies are certainly needed to establish the exact clinical significance of this reduction, changes in myocardial work parameters seem to have a favorable impact on prognosis. Pedersen et al. demonstrated in fact that a 100 mm Hg% increase in GWI pre-TAVI was associated with a 4% lower risk of all-cause mortality [19]; our group also previously demonstrated that baseline GWI is a predictor of all-cause mortality and readmission for heart failure (HF) after TAVI [12]. Similarly, in a larger cohort of 147 TAVI patients, Anwer et al. identified LV GWI as a predictor of cardiovascular mortality [36]. Pedersen et al. also demonstrated a different behavior of LV GWI according to baseline LVEF, with a decrease in GWI only in patients with preserved LVEF and an increase in GWI in patients with reduced LVEF due to increased contractility [19]. Our meta-regression analysis showed that both age and baseline LVEF significantly influenced GWE changes after TAVI. This observation may be explained by several mechanisms; older patients typically present with increased ventricular stiffness, impaired relaxation, and a greater burden of comorbidities, which may reduce the expected improvement in myocardial efficiency despite afterload relief. Baseline LVEF also appears to play a critical role: patients with preserved LVEF often have a compensatory hypercontractile state due to pressure overload, and the relief of afterload may reduce excessive work and improve efficiency. Conversely, in patients with reduced LVEF, the presence of structural myocardial damage and fibrosis may limit the extent of functional recovery, leading to attenuated changes in GWE. Our results can also be interpreted in the broader concept of extravalvular cardiac damage, which recognizes that in severe AS the pathological process extends beyond the valve [37]. The persistence of myocardial fibrosis and irreversible myocyte loss may limit full restoration of contractile function and efficiency, as suggested by the absence of a clear improvement in GWW and GWE. Emerging evidence suggests that adjunctive pharmacological interventions may further support reverse remodeling; SGLT2 inhibitors for instance, have been recently associated with improved outcomes and enhanced LV reverse remodeling in diabetic patients undergoing TAVI [38]. The assessment of non-invasive MW seems to provide key additional information and to reflect with higher accuracy the myocardial performance status; although more evidence is certainly needed in this regard, serial assessment of MW could provide an objective tool to track LV functional recovery, identifying patients who might benefit from adjunctive pharmacological interventions aimed at promoting reverse remodeling.

## 5. Limitations

This study has several limitations. All the studies included in the meta-analysis are cohort studies, and none of them featured a control group. Correlation coefficients and standard deviations were not consistently reported across the included studies. Only Franco et al. [13] reported correlation coefficients, so for the majority of the studies these parameters had to be imputed, potentially introducing systematic bias. The substantial heterogeneity observed for GWI and GCW (I^2^ = 97.0% and 97.4%, respectively) represents a major limitation of this study. Such variability is likely to be multi-factorial, reflecting the nature of the variable analyzed, as well as differences in study design, intervention type, and follow-up duration. In this regard, differences in the timing of post-procedural echocardiographic assessment after TAVI may represent a critical source of heterogeneity. Nevertheless, most studies included in the analysis adopted a relatively short follow-up, ranging from a few days to one month. Although this may not reflect long-term myocardial adaptation after TAVI, it permits us to draw consistent conclusions in the short term. Moreover, sensitivity analyses indicated that no single study accounted for the observed heterogeneity. Lastly, some included studies had relatively small sample sizes, thus increasing the risk of bias.

## 6. Conclusions

TAVI was associated with significant reductions in GWI and GCW, reflecting decreased myocardial workload and improved energetic efficiency following the resolution of LV outflow obstruction. No significant changes were observed in GWW and GWE, suggesting that these parameters may be less sensitive in capturing subtle myocardial changes in this setting. Although these results should be interpreted with caution given the substantial heterogeneity among studies, our findings support the potential utility of myocardial work indices for assessing myocardial response to AS relief. Further large-scale and longitudinal studies are warranted to confirm their prognostic significance and clarify their role in clinical practice.

## Figures and Tables

**Table 1:** Patients’ baseline characteristics.

Study	Sample Size	Age (y)	Male *n* (%)	Hypertension *n* (%)	Diabetes *n* (%)	Dyslipidemia *n* (%)	eGFR at Baseline (mL/min/1.73 m^2^)	Clinically Relevant CAD *n* (%)	Euro-Score II	Mean Follow-Up
De Rosa et al. [12], 2022	73	80.8 ± 6	39 (54%)	69 (94.5%)	17 (23%)	45(62%)	N/A	14 (19%)	N/A	24 months
Franco et al. [13], 2023	53	80.15 ± 2.8	21 (40%)	53 (100%)	26 (49%)	53 (100%)	N/A	30 (57%)	N/A	6 days
Ilardi et al. [14], 2024	95	79.9 ± 6.4	35 (40%)	80 (90.9%)	23 (26%)	51 (58%)	N/A	29 (33%)	5.3 ± 6.0	12 months
Jain et al. [15], 2021	35	81 ± 7.6	18 (51%)	27 (77%)	10 (29%)	24 (69%)	N/A	24 (69%)	N/A	N/A
Ladányi et al. [16], 2024	90	80 [75–84]	50 (56%)	87 (97%)	34 (38%)	N/A	N/A	43 (48%)	11.5 [7.2–18.1]	12 months
Moya et al. [17], 2024	110	83 ± 6	51 (46%)	67 (62%)	33 (31%)	49 (45%)	N/A	N/A	N/A	521 days
Myon et al. [18], 2023	102	84.3 ± 6.2	56 (55%)	69 (68%)	12 (12%)	53 (52%)	83.0 [35.8]	N/A	N/A	22 months
Pedersen et al. [19], 2024 HG, LVEF > 50%	169	80 ± 6	N/A	N/A	N/A	N/A	68 [51–82]	N/A	2.2 [1.6–3.3]	60 months
Pedersen et al. [19], 2024 HG, LVEF < 50%	60	80 ± 4	N/A	N/A	N/A	N/A	60 [42–75]	N/A	3.2 [2.1–6.0]	60 months
Pedersen et al. [19], 2024 LFLG, LVEF < 50%	82	79 ± 8	N/A	N/A	N/A	N/A	56 [46–67]	N/A	4.6 [2.2–8.2]	60 months
Pedersen et al. [19], 2024 LFLG, LVEF > 50%	45	80 ± 7	N/A	N/A	N/A	N/A	62 [51–75]	N/A	2.2 [1.6–3.8]	60 months
Pedersen et al. [19], 2024 NFLG, LVEF < 50%	34	80 ± 7	N/A	N/A	N/A	N/A	64 [46–74]	N/A	3.7 [2.4–8.3]	60 months
Pedersen et al. [19], 2024 NFLG, LVEF > 50%	83	78 ± 9	N/A	N/A	N/A	N/A	72 [60–85]	N/A	2.2 [1.7–3.9]	60 months
Polewczyk et al. [22], 2025	135	79 ± 7	69 (51%)	86 (64%)	49 (36%)	N/A	N/A	86 (64%)	N/A	6 days
Quinio et al. [20], 2023	125	N/A	40 (59%)	N/A	N/A	N/A	N/A	66 (53%)	N/A	7 days
Wu et al. [21], 2024	255	82 [77–85]	130 (51%)	177 (69%)	70 (28%)	133 (52%)	61 ± 22	141(55%)	N/A	59 months

Categorial variables are given as absolute numbers and percentage, *n* (%). Continuous variables are given as mean ± standard deviation or median [IQR, interquartile range]. Legend. CAD: coronary artery disease; eGFR: estimated glomerular filtration rate; HG: high gradient; LFLG: Low-flow low-gradient; LVEF: left ventricular ejection fraction; NFLF: normal-flow low-gradient.

**Table 2:** Main echocardiographic parameters in patients treated with TAVI at baseline.

Study	LVEDVi (mL/m^2^)	LVEF (%)	LVMi (g/m^2^)	LAVi (mL/m^2^)	AV Mean Gradient (mmHg)	AVA (cm^2^)	LVGLS (%)	GWI (mmHg%)	GCW (mmHg%)	GWW (mmHg%)	GWE (%)
De Rosa et al. [12], 2022	N/A	52.8 ±9.3	N/A	43.8 ± 8.4	46.7± 15.8	0.78 ± 0.16	−16.8 ± 5.0	2288.5 ± 905.2	2713.3 ± 114.9	205.0 ± 179.8	90.5 ± 6.7
Franco et al. [13], 2023	N/A	47.7 ± 9.1	N/A	N/A	57.14 ± 12.63	0.38 ± 0.9 *	N/A	2139.7 ± 278.5	2367.17 ± 318.57	286.47 ± 60.79	100.5 ± 14.7
Ilardi et al. [14], 2024	37.9 ± 17.9	58.2 ± 8.5	55.4 ± 15.9	47 ± 14.3	49.3 ± 12.5	0.7 ± 0.2	18.4 ± 4.2	2406 ± 567	2783 ± 616	238 ± 203	90 ± 6
Jain et al. [15], 2021	N/A	58.2 ± 15.7	115.4 ± 37.0	50 ± 17.9	38.4 ± 12.4	0.8 ± 0.2	−14.2 ± 4.3	1425.7 ± 595.8	1586.2 ± 705.1	133.5 ± 109.3	88.9 ± 11.9
Ladányi et al. [16], 2024	60.1 ± 21.9	52.6 ± 13.1	124.91 ± 37.34	45.1 ± 17.2	45.6 ± 19.9	0.71 ± 0.25	−13.5 ± 4.6	1913 ± 799	2365 ± 851	260 ± 158	88.2 ± 7.1
Moya et al. [17], 2024	N/A	51 ± 10	N/A	45 ± 17	50 ± 15	0.6 ± 0.3	−15.7 ± 5.8	2381 ± 785	2940 ± 942	239 ± 191	91± 6
Myon et al. [18], 2023	58.47 ± 23.42	63 [54–70]	111.5 [92.2–139.0]	46.0 [40.2–60.0]	52.29 ± 16.72	0.72 [0.57–0.83]	−14.11 ± 3.88	2099 ± 692	2463 ± 736	214 [149–357]	87.0 ± 5.66
Pedersen et al. [19], 2024 HG, LVEF > 50%	N/A	59 ± 6	N/A	41 [32–50]	51 [45–61]	0.4 ± 0.1 *	−13.6 ± 3.9	2396 ± 521	2723 ± 550	167 [110–254]	91 [89–94]
Pedersen et al. [19], 2024 HG, LVEF < 50%	N/A	48 ± 8	N/A	42 [33–53]	50 [44–64]	0.3 ± 0.1 *	−9.2 ± 3.5	1577 ± 494	1853 ± 536	168 [118–252]	87 [84–92]
Pedersen et al. [19], 2024 LFLG, LVEF < 50%	N/A	43 ± 10	N/A	47 [36–60]	28 [21–32]	0.4 ± 0.1 *	−10.6 ± 3.3	1171 ± 503	1407 ± 533	155 [108–207]	87 [81–90]
Pedersen et al. [19], 2024 LFLG, LVEF > 50%	N/A	57 ± 6	N/A	45 [30–59]	30 [25–36]	0.3 ± 0.1 *	−13.6 ± 3.9	1850 ± 581	2190 ± 590	188 [113–263]	90 [87–93]
Pedersen et al. [19], 2024 NFLG, LVEF > 50%	N/A	59 ± 6	N/A	34 [27–48]	33 [27–37]	0.5 ± 0.1 *	−15.8 ± 3.3	2275 ± 576	2555 ± 590	143 [98–216]	92 [89–95]
Pedersen et al. [19], 2024 LFLG, LVEF < 50%	N/A	40 ± 7	N/A	36 [28–47]	33 [25–36]	0.4 ± 0.1 *	−10.6 ± 3.3	1468 ± 572	1707 ± 648	155 [114–237]	88 [84–93]
Polewczyk et al. [22], 2025	N/A	49.5 ± 12.8	N/A	N/A	N/A	N/A	−10.5 ± 3.8	966.5 ± 456.1	1315.5 ± 487.5	238.8 ± 160.7	81.6 ± 10.5
Quinio et al. [20], 2023	N/A	59.8 ± 13.3	111 [IQR 46.0]	46.0 [IQR 19.9]	52.5 ± 16.1	N/A	−14.0 ± 3.90	2014 ± 714	2379 ± 761	282 ± 175	87.1 ± 5.98
Wu et al. [21], 2024	N/A	56 [45–63]	N/A	N/A	43 [33–53]	0.7 [0.6–0.8]	−13.6 [−11.0–16.1]	1882 ± 767	2248 ± 809	247 [128–300]	89 [84–93]

Categorial variables are given as absolute numbers and percentage, *n* (%). Continuous variables are given as mean ± standard deviation or median [IQR, interquartile range]. * Values refer to AVA index (cm^2^/m^2^). Legend. LVEDVi: left ventricular end-diastolic volume indexed; LVEF: left ventricular ejection fraction; LVMi: left ventricular mass indexed; LAVi: index left atrial volume; AV: aortic valve; AVA: aortic valve area; LVGLS: left ventricular longitudinal strain. GCW, global constructive work; GWE, global work efficiency; GWI, global work index; GWW, global wasted work.

**Table 3:** Grading of evidence for the association of MW parameters in patients treated with TAVI.

Certainty Assessment							Certainty
Number of Studies	Study Design	Risk of Bias	Inconsistency	Indirectness	Imprecision	Other Considerations	
GWI							
11	Retrospective and prospective cohort studies	Serious concern	Serious concern	Serious concern	Some concern	No publication bias	Very low
GWE							
11	Retrospective and prospective cohort studies	Serious concern	Serious concern	Serious concern	Some concern	No publication bias	Very low
GCW							
11	Retrospective and prospective cohort studies	Serious concern	Serious concern	Serious concern	Some concern	No publication bias	Very low
GWW							
11	Retrospective and prospective cohort studies	Serious concern	Serious concern	Serious concern	Some concern	No publication bias	Very low

## Data Availability

The data underlying this article will be shared on reasonable request to the corresponding author.

## Supplementary Materials

The following supporting information can be downloaded at: https://www.mdpi.com/article/10.3390/jcm14196997/s1, Table S1: PRISMA Checklist; Table S2: Quality assessment for studies conducted with the Newcastle Ottawa Scale; Table S3: Risk of bias assessment for the 11 studies included in the meta-analysis; Table S4: Leave one-out analyses for non-invasive myocardial work outcomes.

## Abbreviations

The following abbreviations are used in this manuscript:

AS	Aortic stenosis
CI	Confidence interval
GCW	Global constructive work
GWE	Global work efficiency
GWI	Global work index
GWW	Global wasted work
HF	Heart failure
LV	Left ventricle
LVEF	Left ventricular ejection fraction
MW	Myocardial work
SD	Standard deviation
TAVI	Transcatheter aortic valve implantation
