# Supplementary material for: Effect of Transcatheter Aortic Valve Implantation on Non-Invasive Myocardial Work Parameters: A Systematic Review and Meta-Analysis
Source: J Clin Med. 2025 Oct 2;14(19):6997. doi: 10.3390/jcm14196997 (PMC12524723; doi:10.3390/jcm14196997)
Supplement: Supplementary file 1 [file jcm-14-06997-s001.zip › Table S2_Quality assessment for studies conducted with the Newcastle Ottawa Scale;.pdf]

The Newcastle-Ottawa Scale (NOS) for assessing the quality of studies in meta-analyses.

**Studies and year**	**Selection**				**Comparability**		**Outcomes**		**Total**
	**Representativeness of the exposed cohort**	**Selection of the non exposed cohort**	**Ascertainment of exposure**	**Demonstration that outcome of interest was not present at start of study**	**Compare ability of cohorts on the basis of the design or analysis**	**Assessment of outcome**	**Was follow-up long enough for outcomes to occur**	**Adequacy of follow up of cohorts**	
De Rosa et al., 2022	★	☆	★	★	★★	★	★	★	★★★★★★★★☆
Franco et al., 2023	★	☆	★	★	★★	★	★	☆	★★★★★★★☆☆
Ilardi et al., 2024	★	☆	★	★	★★	★	★	★	★★★★★★★★☆
Jain et al., 2021	☆	☆	★	★	★★	★	☆	☆	★★★★★☆☆☆☆
Ladányi et al., 2024	★	☆	★	★	★★	★	★	★	★★★★★★★★☆
Moya et al., 2024	★	☆	★	★	★★	★	★	★	★★★★★★★★☆
Myon et al., 2023	★	☆	★	★	★★	★	★	★	★★★★★★★★☆
Pedersen et al., 2024	★	☆	★	★	★★	★	★	★	★★★★★★★★☆
Polewczyk et al., 2025	★	☆	★	★	★★	★	★	☆	★★★★★★★☆☆
Quinio et al., 2023	★	☆	★	★	★★	★	★	☆	★★★★★★★☆☆
Wu et al., 2024	★	☆	★	★	★★	★	★	★	★★★★★★★★☆
