# Supplementary material for: Effect of Transcatheter Aortic Valve Implantation on Non-Invasive Myocardial Work Parameters: A Systematic Review and Meta-Analysis
Source: J Clin Med. 2025 Oct 2;14(19):6997. doi: 10.3390/jcm14196997 (PMC12524723; doi:10.3390/jcm14196997)
Supplement: Supplementary file 1 [file jcm-14-06997-s001.zip › Table S4.pdf]

Supplementary Table 4. Grading of evidence for the association of myocardial work parameters in patients treated with TAVI.

Certainty assessment							Certainty
**Number of studies**	**Study design**	**Risk of bias**	**Inconsistency**	**Indirectness**	**Imprecision**	**Other considerations**	
**GWI**							
11	Retrospective and prospective cohort studies	Serious concern	Serious concern	Serious concern	Some concern	No pubblication bias	Very low
**GWE**							
11	Retrospective and prospective cohort studies	Serious concern	Serious concern	Serious concern	Some concern	No pubblication bias	Very low
**GCW**							
11	Retrospective and prospective cohort studies	Serious concern	Serious concern	Serious concern	Some concern	No pubblication bias	Very low
**GWW**							
11	Retrospective and prospective cohort studies	Serious concern	Serious concern	Serious concern	Some concern	No pubblication bias	Very low
