# Supplementary figures and images for: Effect of Transcatheter Aortic Valve Implantation on Non-Invasive Myocardial Work Parameters: A Systematic Review and Meta-Analysis
Source: J Clin Med. 2025 Oct 2;14(19):6997. doi: 10.3390/jcm14196997 (PMC12524723; doi:10.3390/jcm14196997)

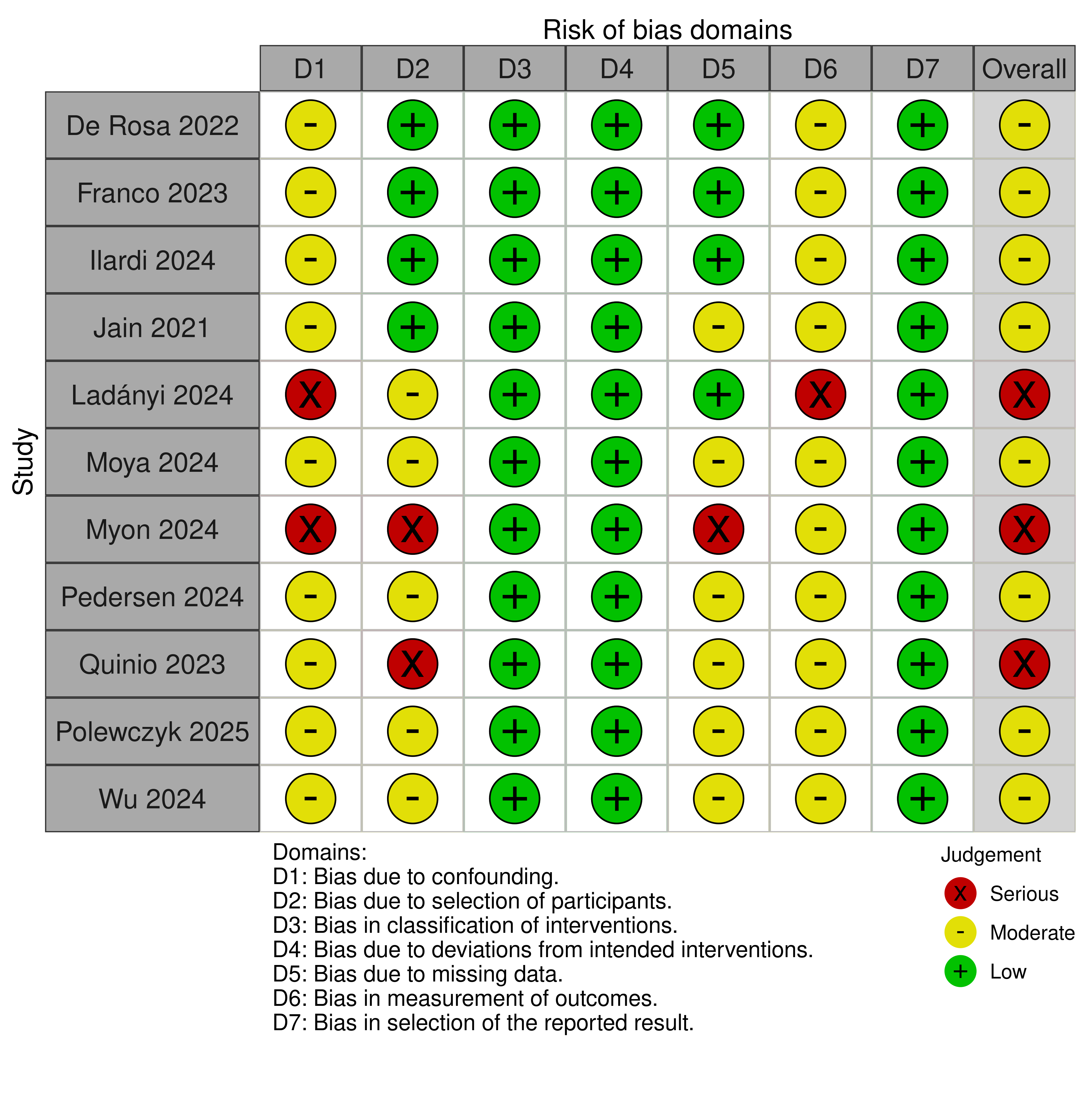

Supplement: Supplementary file 1 [file jcm-14-06997-s001.zip › Table S3.png]
